# Novel Approaches Examining Sleep Health as a Marker of Successful Aging

**DOI:** 10.1093/geroni/igab046.1303

**Published:** 2021-12-17

**Authors:** Soomi Lee, Meredith Wallace

## Abstract

Sleep is a modifiable determinant of health. It changes with advancing age and in response to diverse contexts (e.g., related to work or one’s health). Previous studies have often used single measures of sleep duration or sleep quality. However, a recent paradigmatic shift towards multidimensional sleep health emphasizes the importance of examining how multiple sleep measures are simultaneously associated with health. This approach presents many opportunities for understanding sleep phenotypes and their potential contributions to health. Yet it also presents methodological challenges in analyzing multidimensional sleep data. This symposium showcases the most recent approaches and novel ideas examining the role of sleep health in successful aging. Paper 1 examines sleep profiles (i.e., latent groups with varying sleep characteristics) in middle-aged adults and their linkages to psychological well-being and chronic conditions with differences by age groups. Paper 2 investigates 24-hour patterns of sleep-activity rhythms and their associations with physical functioning performance in older men and women. Paper 3 showcases the utility of a sleep health composite score in examining sleep disparities and their drivers in middle- and later-adulthood. Paper 4 examines whether and how a composite sleep health measure based on actigraphy data is associated with specific characteristics of adult bipolar disorder patients. These papers use different cohorts, such as the Midlife in the United States Study, Osteoporotic Fractures in Men study, and Multi-Ethnic Study of Atherosclerosis. At the end, Dr. Wallace will discuss key findings from these studies, their methodological contributions and implications for aging, and directions for future research.

